# CAFs orchestrates tumor immune microenvironment—A new target in cancer therapy?

**DOI:** 10.3389/fphar.2023.1113378

**Published:** 2023-03-14

**Authors:** Chunxue Zhang, Yuxiang Fei, Hui Wang, Sheng Hu, Chao Liu, Rong Hu, Qianming Du

**Affiliations:** ^1^ School of Basic Medicine and Clinical Pharmacy, China Pharmaceutical University, Nanjing, China; ^2^ Department of Pharmacy, Nanjing First Hospital, Nanjing Medical University, Nanjing, China; ^3^ College of Pharmacy, Xinjiang Medical University, Urumqi, China; ^4^ State Key Laboratory of Natural Medicines, Department of Physiology, China Pharmaceutical University, Jiangsu Nanjing, China; ^5^ General Clinical Research Center, Nanjing First Hospital, Nanjing Medical University, Nanjing, China

**Keywords:** tumor microenvironment, cancer-associated fibroblasts, immune cells, CAF-targeted therapy, cancer

## Abstract

Cancer immunotherapy has opened a new landscape in cancer treatment, however, the poor specificity and resistance of most targeted therapeutics have limited their therapeutic efficacy. In recent years, the role of CAFs in immune regulation has been increasingly noted as more evidence has been uncovered regarding the link between cancer-associated fibroblasts (CAFs) and the evolutionary process of tumor progression. CAFs interact with immune cells to shape the tumor immune microenvironment (TIME) that favors malignant tumor progression, a crosstalk process that leads to the failure of cancer immunotherapies. In this review, we outline recent advances in the immunosuppressive function of CAFs, highlight the mechanisms of CAFs-immune cell interactions, and discuss current CAF-targeted therapeutic strategies for future study.

## 1 Introduction

Cancer, a major global public health problem, is the second leading cause of death (Deo et al., 2022). The process of cancer progression is accompanied by dynamic changes in the microenvironment, forming the tumor microenvironment (TME) (Duan et al., 2020). TME is capable of inducing tumor immunosuppression, metastasis, drug resistance, and response to targeted therapies, which is one of the major causes of cancer treatment failure (Bejarano et al., 2021). The TME is a highly complex, dynamically evolving, finely regulated system composed of tumor cells, infiltrating immune cells, cancer-associated fibroblasts (CAFs), endothelial cells, lipid cells, extracellular matrix (ECM), and multiple signaling molecules. CAFs, a major component of TME, have been shown to originate from various cellular precursors types, such as tissue-resident fibroblasts, bone marrow mesenchymal stem cells, and pericytes (Hinshaw and Shevde, 2019). Numerous studies have demonstrated that CAFs promote tumor cell proliferation, drug resistance, and invasive metastasis by participating in a variety of biological processes, and are involved in exogenous pathways such as angiogenesis, ECM remodeling, and epithelial-mesenchymal transition (Feng et al., 2022). Therefore, understanding the nature of CAFs and their functional mechanisms in cancer development is beneficial for finding new cancer treatment strategies.

A growing body of clinical evidence demonstrates the superior efficacy of cancer immunotherapy in many tumor types (Riley et al., 2019). Modulation of the immune system by immune checkpoint inhibitors (ICIs), such as anti-CTLA4, has led to remission in a wide range of tumors (Riley et al., 2019). Despite these successes, immunotherapy still has inevitable limitations for most cancer patients, such as drug resistance and limited applicability (O'Donnell et al., 2019). Studies have shown that the complexity and diversity of the immune environment of the tumor microenvironment have important implications for immunotherapy (Barrett and Puré, 2020; Kockx et al., 2021). As major components of the tumor immune microenvironment, infiltrating immune cells can be both pro-tumor (Tumor-associated macrophages, TAM) and anti-tumor (Cytotoxic CD8^+^ T cells), and the ratio of these two populations play an important role in tumor progression (Lin et al., 2019; Woan and Miller, 2019). Therefore, the tumor immune microenvironment (TIME) plays a key role in cancer immunotherapy. Numerous studies indicate that CAFs regulate immune infiltration and affect TIME composition, and influence the outcome of cancer immunotherapy (Barrett and Puré, 2020). CAFs control the infiltration and phenotypic alterations of immune cells as well as influence their spatial movement within the tumor to promote immune regulation (Xiang et al., 2022). For example, CAFs can induce immunosuppressive cells such as regulatory T cells (Treg cells) and myeloid-derived suppressor cells (MDSCs) by secreting various cytokines including CXCL12 and other effector molecules, suppressing the immune function of immune effector cells and cytotoxic T lymphocytes ultimately create a TIME for immune tolerance that is conducive to tumor progression (Mao et al., 2021). Therefore, understanding the interactions between CAFs and immune cells can identify the mechanism of immunosuppression of CAFs more accurately, thus discovering effective therapy targeting CAFs, which can improve the success rate of immunotherapy. This review describes the research progress on immunosuppressive mechanisms mediated by CAFs from the perspective of CAFs-immune cell interactions in TME, concludes the major CAF-based targeted immunotherapy technologies, and discusses the shortcomings of current CAF-targeted therapies for future study.

## 2 Origins and activators of CAFs

**FIGURE 1:**
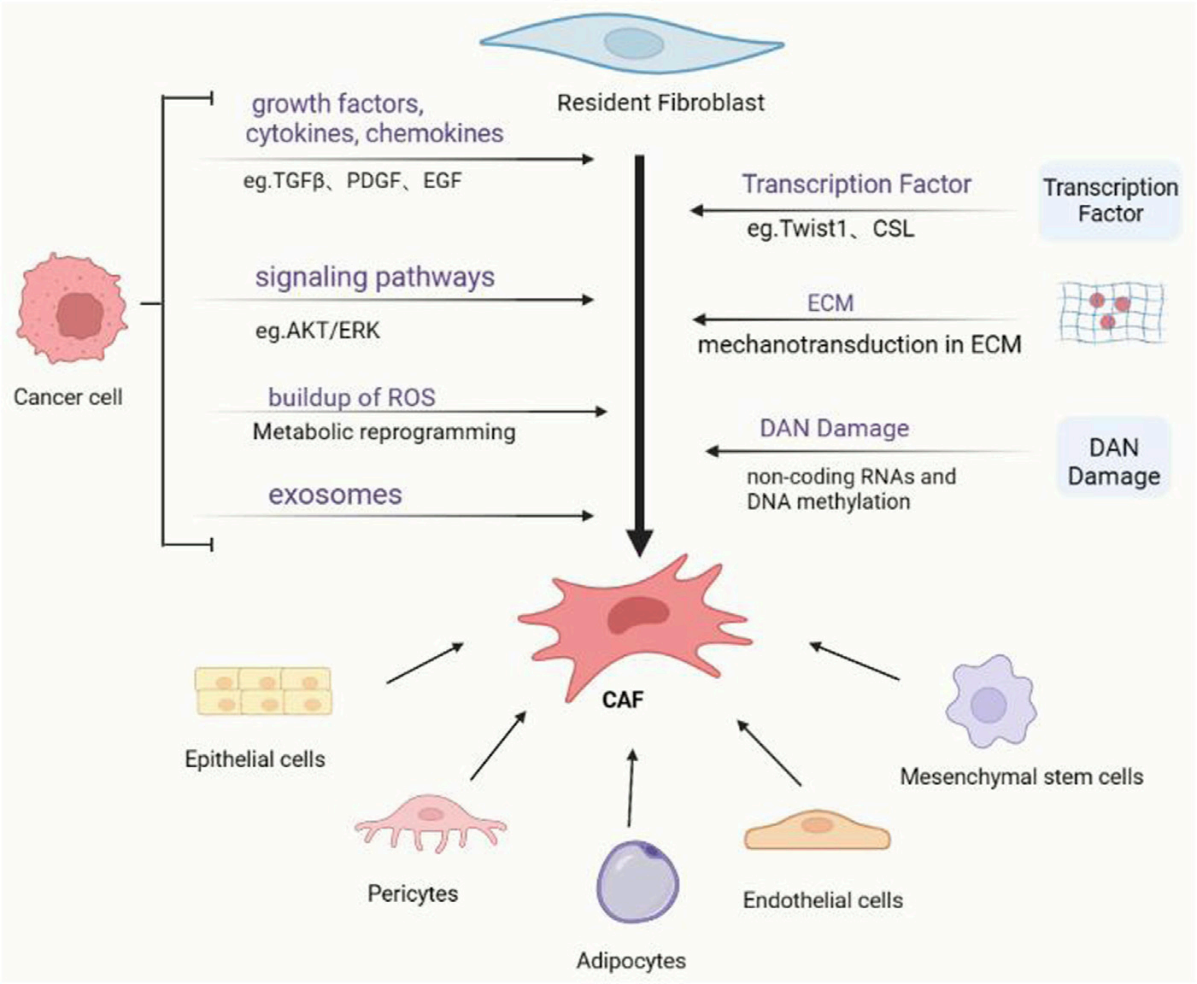
Cancer-associated fibroblasts (CAFs) and their activation mechanisms. Various progenitor cells can give rise to cancer-associated fibroblasts *via* diverse pathways. The most researched process is the activation of local fibroblasts, which can be triggered by a variety of triggers, including tumor-secreted proteins and physical TME features.

The origin of CAFs is difficult to determine because of the lack of unique biomarkers that are not expressed in any other cells (Figure 1). Most scientists believe that fibroblasts are derived from primitive mesenchymal cells, whereas CAFs are derived from activated fibroblasts in local tissues (Arina et al., 2016). CAFs have also been found to originate from other cells, such as mesenchymal stem cells (MSCs), epithelial cells, pericytes, adipocytes, and endothelial cells (Simon and Salhia, 2022).

CAFs in the tumor stroma can be identified by their shape as well as specific identification markers. The production of a-smooth muscle (α-SMA) is commonly used to activate cancer-associated fibroblasts (Nurmik et al., 2020). a-SMA is the most commonly used maker for identifying CAFs (Nurmik et al., 2020). Fibroblast activation protein (FAP) is a membrane protein that is expressed specifically in CAFs induced by many types of human epithelial carcinoma cells (Chen and Song, 2019). CAFs contain the proteins fibroblast-specific protein 1 (FSP1), vimentin, and platelet-derived growth factor receptor (PDGFR) (Chen and Song, 2019). They could be indicators of cancer-related fibrillogenic cell activity.

Tissue-resident fibroblasts are typically activated by cytokines secreted by tumor cells and other stromal cells, such as specifically transforming growth factor beta (TGFβ), platelet-derived growth factor (PDGF), basic fibroblast growth factor (bFGF), epithelial growth factor (EGF), connective tissue growth factor (CTGF), hepatocyte growth factor (HGF) and vascular endothelial growth factor (VCAM-1) (Mao et al., 2021). TGFβ is a multifunctional cytokine that affects cell proliferation, differentiation, and migration. It is thought to be the most efficient cytokine for eliciting CAF activation. TGFβ activates the TGF-β/Smad classical pathway directly, decreasing αSMA expression and increasing contractile cytoskeleton activity (Liu et al., 2016). Resident fibroblasts can stimulate CAF transformation by directly activating the TGFβ-/Smad classical pathway *via* autocrine TGF-β to regulate *a*-SMA expression (Peng et al., 2022). Protein kinase B (AKT) and extracellular signal-regulated kinase (ERK) pathways activation can also influence *a*-SMA expression (Kuzet and Gaggioli, 2016; Foglia et al., 2019). TGFβ can also indirectly activate Smad2-mediated TGF-β-driven transformation from fibroblasts to CAFs in gastric cancer *via* upregulation of lactose lectin-1 (galectin-1, Gal1) expression in fibroblasts *via* the PI3K/Akt axis (Zheng et al., 2016). Chloride intracellular channel 4 (CLIC4) is a protein that highly upregulated during TGF-β-induced fibroblasts differentiation into activated fibroblasts and is thought to be important in the TGF-β signaling pathway (Shukla et al., 2014). The specific mechanism is that TGF-β promotes fibroblast transformation through CLIC4-mediated p38 map kinase activation upregulating the expression of CAF markers (Shukla et al., 2014).

MSCs transdifferentiate into CAFs through activating tumor cell and stromal cell-secreted transforming growth factor beta-1 (TGFβ-1) and C-X-C chemokine ligand (CXCL) 16 (Jung et al., 2013; Barcellos-de-Souza et al., 2016; Jiang et al., 2016; Ganguly et al., 2020). MSCs generated from bone marrow can be transformed into myofibroblasts in mouse fibrotic liver *via* the sphingosine kinase/sphingosine 1 phosphate receptor axis by TGF-β1 (Yang et al., 2012; Chandra Jena et al., 2021). When activated with TGFβ-1, human adipose tissue-derived stem cells (HASCs) transdifferentiate into CAFs with a fibroblastic phenotype (α-smooth muscle actin and tenascin-C expression) (Jotzu et al., 2011; de Araújo Farias et al., 2018). However, less evidence supports these origins, and their relevance to other types of tumors is limited.

In addition to the regulatory molecules mentioned above, inflammatory cytokines in TME such as chemokines (CXL), interleukins (IL), interferons (IFN), and tumor necrosis factors (TNF) not only govern CAF activation but also directly or indirectly promote tumor growth (Rollins, 2006; Mhaidly and Mechta-Grigoriou, 2020). TNF stimulates CAFs to produce C-C motif chemokine ligand2 (CCL2), C-C motif chemokine ligand 5 (CCL5), C-C motif chemokine ligand 7 (CCL7), C-X-C motif chemokine ligand 8 (CXCL8), C-X-C motif chemokine ligand 12 (CXCL12), C-X-C motif chemokine ligand 14 (CXCL14), and C-X-C motif chemokine ligand 16 (CXCL16), which activate ERK and AKT signaling pathways through focal mucin kinase (FAK), increasing the transcriptional activity of downstream -catenin and NF-κB. Interleukin-1 (IL-1), interleukin-6 (IL-6), interleukin-8 (IL-8), or IFN interact with receptors on the surface of CAFs such as IL6-R and CXC receptor 2 (CXCR2). CAFs express IL6-R, CXCR2, and other receptors that activate Janus kinase/signal transducer and activator of transcription (JAK1/STAT3), Rho-associated kinases (ROCK), or AKT/ERK1/2 signaling pathways to activate CAFs, increase myosin contractility, and ECM (Prabhavathy et al., 2014; Kuzet and Gaggioli, 2016). Many transcription factors, including Notch signaling transcriptional repressor (CBF1/RBP-J/suppressor of hairless/LAG-1, CSL) and recombinant human activating transcription factor-3 (ATF3), can operate as mediators of CAF activation (Kim et al., 2017). Twist-related protein 1 (Twist1) and paired related homeobox 1 (Prrx1) are specific transcription factors that positively regulate CAFs activation. The study confirmed that Twist1-Prrx1-TNC can form a positive feedback loop to induce the activation of CAFs (Yeo et al., 2018). In addition, CD44, a cell surface molecule expressed by MSCs, can also induce the activation of CAFs by up regulating Twist transcription (Spaeth et al., 2013; Hu et al., 2022). Myristoylated alanine-rich protein kinase C substrate (MARCKS), the forkhead box F1 gene (FoxF1), and the zinc finger transcription factor (snail 1) by activating AKT/Twist1 signaling to upregulate αSMA and PDGFR, the release of paracrine factors such as FGF-2 and HGF is promoted, resulting in the activation of CAFs and tumor (Saito et al., 2010; Stanisavljevic et al., 2015; Yang et al., 2016a).

The transformation of local fibroblasts into CAFs is also triggered by the buildup of reactive oxygen species (ROS). By increasing the expression of growth factors such as PDGF and TGF-β, ROS regulates the communication between cancer cells and fibroblasts, subsequently triggering the release of chemokines such as CXCL12 (Costa et al., 2014). Additionally, CAF activation is influenced by radiation therapy-induced DNA damage, tumor-derived exosomes, and ECM (Calvo et al., 2013; Hellevik and Martinez-Zubiaurre, 2014; Giusti et al., 2018).

## 3 CAF-mediated regulation of the innate anti-tumor immune response

### 3.1 Interaction between CAFs and tumor-associated macrophages

Tumor-associated macrophages (TAMs) are heterologous cell populations with distinct functional phenotypes due to their flexibility; they are classified as M1 or M2 based on their functional differentiation state and immune response (Arvanitakis et al., 2022). M1 types are involved in T helper type 1 (Th1) cells responses, activated by cytokines such as interferon-gamma (IFN-γ), tumor necrosis factor (TNF-α), or lipopolysaccharide (LPS), and are characterized by the secretion of pro-inflammatory molecules and reactive ROS (Grivennikov et al., 2010; Yang et al., 2020). M2 types are involved in T helper type 2 (Th2) cells immune responses which promote tissue repair, angiogenesis promotion, immunosuppressive factors secretion, inhibit cytotoxic T cell killing, and promote tumor cell invasion and metastasis and ECM remodeling (Grivennikov et al., 2010; Yang et al., 2020). Now, some studies show that TAM cells rarely show true M1 or M2 phenotypes (Locati et al., 2020; Mao et al., 2021). This means that this binary classification is not a good way to understand how complicated these cells are. Some researchers try to get around this problem by putting macrophages into different groups (like M2a, M2b, and M2c instead of M2) or by using more general terms (such as M1-like and M2-like, rather than M1 and M2) (Szulc-Kielbik and Kielbik, 2012). But making changes to these definitions might not be enough to cover all of the complexity of TAM.

CAFs and TAMs are essential components of the tumor microenvironment, coordinating pro-tumor inflammation (Tajaldini et al., 2022). TAMs are the most abundant innate immune cell type in the vicinity of densely populated areas of CAF, suggesting a close relationship between TAMs and CAFs (Gunaydin, 2021). In addition to their roles in monocyte recruitment and TAM differentiation, there are numerous studies suggesting that the interaction between CAFs and TAMs promotes cancer growth and the activation of immunosuppressive macrophages to generate an immunosuppressive environment (Raskov et al., 2021). CAFs and M2-polarized macrophages play a synergistic role in the progression of prostate cancer, as evidenced by an examination of patients with prostate cancer at various clinical stages (Comito et al., 2014). A similar pattern of CAF-promoted macrophage recruitment and TAM differentiation for cancer progression has also been found in other cancer types. In pancreatic ductal adenocarcinoma (PDAC), CAFs were able to produce the TAM phenotype in part *via* released M-CSF and incerased ROS generation in monocytes, and CAFs-induced M2 macrophages greatly promoted the proliferation, migration, and invasion of pancreatic tumor cells (Zhang et al., 2017). In colorectal cancer, Haaglim Cho et al. demonstrated that CAF production of GM-CSF and IL6 in response to cancer cell stimulation induced human monocytes to differentiate into pro-invasive M2-like macrophages (Yeo et al., 2018). Andersson et al. discovered that CAFs induce TAM phenotypic transition from M1 to M2 by releasing high amounts of interleukin-33 (IL-33), which leads to cancer metastasis *via* the IL-33/NF-Bκ/MMP9/laminin axis (Andersson et al., 2018). In oral squamous cell carcinoma (OSCC), XingLia et al. found that CAFs stimulates monocyte differentiation into M2 macrophages *via* the CXCL12/CXCR4 pathway (Li et al., 2019). These polarized M2 macrophages then promoted the formation of CSC-like cells in OSCC, leading to increased proliferation and decreased apoptosis in OSCC(57). *In vitro* investigations conducted by Shuhai Chen and colleagues revealed that CAF enhances macrophage M2 polarization and CAF-secreted CXCL12 causes TAMs to release plasminogen activator inhibitor-1 (PAI-1), hence promoting HCC progression (Chen et al., 2021). Ran Zhang et al. found that knocking down G protein-coupled receptor 30 (GPR30) in CAFs reduced CXCL12 expression and thus inhibited macrophage migration, as well as that macrophages had attenuated M2 polarization, downregulated M2-like marker expression, and inhibited prostate cancer (PCa) cell invasion by reducing IL-6 secretion (Zhang et al., 2021). Furthermore, CAF-derived Chitinase 3-like 1, associated with inflammatory disease, has been reported to contribute to tumor growth in breast cancer with high infiltration of M2-polarised macrophages and TH2-type immune responses (Cohen et al., 2017). C-C chemokine ligand 2 (CCL2), derived from FSP1 CAF enhances inflammatory response in skin tumors by increasing monocyte recruitment, which promotes cancer development (Zhang et al., 2011). More importantly, CAFs may be associated with the establishment of an immunosuppressive environment through the induction of immunosuppressive macrophages. The current study found a significant relationship between the number of TAMs and the CAFs grade in breast cancer (Gok Yavuz et al., 2019). High grade CAF tissues contained more CD163 or CD206 macrophages, according to research by Betul Gok Yavuz and colleagues (Gok Yavuz et al., 2019). Additionally, fewer CD163 or CD206 macrophages were linked to low CAF grade (Gok Yavuz et al., 2019). Betul Gok Yavuz et al.(2019) also identified the role of CAFs on monocyte recruitment and macrophage polarisation in breast cancer and induced immunosuppressive PD-1^+^ TAM to shape the tumor microenvironment. CAF attracts monocytes in colorectal cancer by secreting IL-8 and promotes the polarization and recruitment of macrophage M2. M2 polarized macrophages collaborate with CAF to inhibit natural killer (NK) cell function and protect CRC cells from NK cell-mediated killing (Zhang et al., 2019). In a recent article, the authors demonstrated that co-culture with triple-negative breast cancer (TNBC)derived CAFs resulted in the reprogramming of blood monocytes to immunosuppressive STAB1^+^TREM2^high^ lipid-associated macrophages (LAM), thereby inhibiting T cell activation and proliferation, thereby inducing an immunosuppressive microenvironment (Timperi et al., 2022). Next, CAFs derived from (lung squamous cell carcinoma) LSCC were shown to promote CCR2^+^ monocyte migration, prompting their transformation into immunosuppressive myeloid-derived suppressor cells (MDSCs), and decrease CD8^+^ T cell proliferation and IFN production. The immunosuppressive role of CAF-induced MDSCs might be reversed by targeting CCR2 to reduce monocyte migration and IDO1 or NOX to inhibit ROS generation, hence disrupting CAF-monocyte interactions (Xiang et al., 2020). Furthermore, some studies have shown that M2 TAMs can activate CAFs, hence promoting tumor growth. Macrophage-determined components may promote the transformation of resident hepatic stellate cells into myofibroblasts, resulting in a fibrotic environment in pancreatic ductal adenocarcinoma liver metastases (Nielsen et al., 2016). Osteopontin (OPN) has been identified as a crucial molecule involved in CAF and TAM interactions in HCC. TAMs in the TME release OPN, a chemokine-like phosphorylated glycoprotein. TAM-secreted OPN promotes OPN secretion from CAFs, which increases cancer cell malignancy by upregulating proliferation, ECM degradation, and migration (Tokuda et al., 2021). Additionally, TAM is capable of influencing CAF. It has been observed that TAMs promote neuroblastoma growth by inducing the proliferation and invasion of CAF-like bone marrow mesenchymal stem cells. Activated CAF also increase TAM activity, establishing a positive feedback loop that promotes cancer growth and an immunosuppressive microenvironment (Hashimoto et al., 2016).

Among the different cells present in TME, TAMs, and CAFs have been shown to operate the tissues that make up the ECM to regulate angiogenesis, tumor metastasis, and drug resistance with significant value (Monteran and Erez, 2019). It has been demonstrated that TAM and CAF in the TME establish a barrier with the ECM to isolate the action of drugs and even inhibit the killing action of immune cells, resulting in a poor prognosis and the failure of chemotherapeutic drug treatment (Tajaldini et al., 2022). Given the synergistic relationship between CAFs and TAMs in the TME in promoting cancer development, strategies to alter the polarization phenotype of TAMs should also consider the synergistic effects of CAFs, thereby contributing to a more effective antitumor immune response. Accordingly, the co-focusing of CAFs and TAMs is viewed as a choice to be thought of.

### 3.2 Interaction between CAFs and tumor-associated neutrophils (TANs)

Neutrophils were initially considered the first responders of the innate immune system against extracellular pathogens (Lehrer et al., 1988; Schoen et al., 2022). However, current evidence suggests that neutrophils are not only involved in the regulation of the innate and adaptive immune systems but can polarize toward different phenotypes in response to environmental signals (Hedrick and Malanchi, 2022). On the one hand, N1 TANs exhibit antitumor activity mediated by direct or indirect tumor cell lysis, while on the other hand, N2 TANs have a prominent role in growth, invasion, angiogenesis, and metastasis in various cancer types (Hedrick and Malanchi, 2022). TGF-β plays an important role in neutrophil proliferation plasticity, driving the acquisition of the N2 phenotype (Piccard et al., 2012; Arvanitakis et al., 2021; Mahmud et al., 2022). Additionally, the N2 phenotype is linked to the production of neutrophil extracellular traps (NETs), complexes made of DNA and granule proteins that are released in response to stimulation. NETs contain proteins including matrix metallopeptidase-9 (MMP-9) and histonectin G that promote tumor growth (Brinkmann et al., 2004; Jaillon et al., 2007; Demkow, 2021; Segal et al., 2022).

According to the current report, CAFs may be involved in the recruitment polarization of TANs and induce the immunosuppressive properties of TANs. For instance Cancer-associated fibroblasts infiltrating HCC(HCC-CAFs) recruit peripheral blood neutrophils by the SDF1a/CXCR4 pathway. CAFs infiltrating HCC induce TANs survival and activation, as reflected by increased expression of CD66b, programmed death ligands 1 (PD-L1), IL-8, TNF, and C-C chemokine ligand 22 (CCL22) and decreased expression of CD62L. Also, HCC-CAFs cause PDL1^+^ neutrophils formation through the IL6 signaling transducer and transcriptional activator STAT3-PD-L signaling cascade (Cheng et al., 2018). This stops T cells from working and makes it easier for HCC to spread. Several other studies have also shown that CAFs may be involved in the polarization of TANs. It has been demonstrated that the CAF-expressed cytokine receptor CXCR2 is essential for the recruitment of neutrophils into malignancies. It is possible that CAFs may help TANs move in a way that depends on CXCR2 (Li et al., 2015). In a recent study, Song et al. (2021) investigated the interrelationship between CAFs, HCC cells, and TANs, which is mediated by a cytokine network. The scientists found that the cardiotrophin-like cytokine factor 1 (CLCF1)-CXCL6/TGFβ-axis, as well as the contemporaneous recruitment of N2 TANs, had a role in the control of cancer stemness in a cohort of HCC clinical samples, leading to the poor prognosis of HCC patients. CLCF1, which was made by CAFs, caused tumor cells to make more CXCL6 and TGF-β, which worked on tumor cells to make them more stem-like and on TANs to cause N2 polarization. Next, CAFs were found to induce neutrophil extracellular traps modulates tumor growth. Munir et al. (2021) discovered that neutrophils are frequently limited to CAF-rich areas in primary mouse pancreatic and skin (melanoma) cancers, hinting that there may be crosstalk between the two populations. Through a ROS-dependent mechanism, CAF-secreted amyloid promotes the formation of tumor-associated NETs (t-NETs) *via* CD11b (Munir et al., 2021). Inhibiting the treatment of t-NETs or reducing the generation of amyloid by CAFs can halt tumor growth and restore the antitumor status of neutrophils invading the tumor (Munir et al., 2021). In addition, gastric cancer mesenchymal stem cells (GC-MSCs) and neutrophils interact bidirectionally, according to Zhu et al. (2014) GC-MSCs can stimulate neutrophil chemotaxis and activation through IL-6-mediated STAT3-ERK1/2 axis and activated TANs can promote MSCs becoming CAFs.

In conclusion, CAF may be involved in the recruitment of polarized TANs and the induction of immunosuppressive properties, but the number of articles on the interaction of CAF with TANs is limited, and the specific mechanism of CAF-TAN interaction is not well understood and needs further study.

### 3.3 Interaction between CAFs and natural killer (NK) cells

There are various types of NK cells. Humans have two main subtypes: CD56^bright^CD16^dim^ and CD56^dim^CD16^bright^. The latter subtype, mature NK cells, is more cytotoxic to its targets than the former (Ran et al., 2022). CD56 is a recognized NK cell marker. CD16 is a marker of activated and mature NK cells (Ran et al., 2022). Whether NK cells are active is determined by the expression of activating or inhibitory receptors on the cell surface.

CAFs reduce NK cells activation receptor, IFN-γ, TNF-α, perforin and granzyme B expression by secreting various cytokines, chemokines and MMPs to reduce NK cell toxicity (Li et al., 2013; Huang et al., 2019). For example, MMP secreted by CAFs in the melanoma microenvironment reduced the expression of NKG2D ligand (MICA/B) in NK cells, which further inhibited the killing of tumor cells by NK cells (Ziani et al., 2017). CAFs secrete IDO or PGE2 to reduce NKG2D expression in NK cells, creating an unresponsive state in antitumor immunity (Balsamo et al., 2009; Li et al., 2012). Substantial studies have proven that TGFβ can reduce the cytotoxic actions of NK cells by decreasing the expression of the activating receptors NKG2D, NKp30, and NKp44 on NK cells (Trotta et al., 2008; Ziani et al., 2017; Han et al., 2018; Lim et al., 2019). By controlling the expression of the ligands associated with NK cell activation receptors on tumor cells, CAFs can also subtly inhibit the activity and functionality of NK cells. A decrease in poliovirus receptor (PVR, a ligand of an NK-activating receptor) expression on the cell surface is essential for the CAF-mediated inhibition of NK cells killing activities. Meanwhile, NK cell-mediated cleavage of CAFs was observed in a variety of tumor types. Pancreatic stellate cells (PSCs) are CAFs subpopulations found in PDAC (94). Through interactions between NKG2D and MICA/B, NK cells can target activated PSCs and mediate PSCs lysis (Bachem et al., 2005). However, only a few studies have examined the impact of NK cells on CAFs, and more research is required to clarify how this interaction develops.

### 3.4 Interaction between CAFs and mast cells (MCs)

Mast cells, which develop from CD34/CD117 pluripotent hematopoietic stem cells, are tissue-resident sentinel cells that, upon activation, produce a vast array of chemokines and cytokines. MC1 (meaning anti-tumorigenic) produce granzyme B, IL-9, and histamine, which induce dendritic cells (DCs) maturation and inhibit murine tumor growth. MC2 (meaning pro-tumorigenic) produces VEGFs, FGF, MMP-9, TGF-β, and cytokines (IL-1, IL-6, and IL-13) (Varricchi et al., 2019).

Mast cells and stellate cells, a CAF precursor, have complex relationships. *In vitro*, Ma et al. (2013) discovered that PSC can stimulate and proliferate mast cells. Mast cells, on the other hand, can stimulate CAF synthesis in the TGF-β2-STAT6 non-dependent route by secreting IL-13 and trypsin-like proteases. In breast cancer, MCs promote ECM destruction and myofibroblast differentiation through MMP and trypsin secretion, thus contributing to tumor aggressiveness and metastatic spread (Mangia et al., 2011). Mast cell-derived trypsin indirectly enhances CAF-induced morphological changes in prostate epithelium *via* the tumor microenvironment (Pereira et al., 2019). CAFs recruit mast cells. Gene microarray analysis identified CXCL12 as the primary estrogen-driven target gene in CXCL12, while CAFs recruited mast cells in a CXCR4-dependent manner *via* CXCL12 (Ellem et al., 2014). To date, the interactions between mast cells and CAFs in TME are still poorly understood and need further study.

### 3.5 Interaction between CAFs and dendritic cells (DCs)

Among all immune cells, DCs are the most potent antigen-presenting cells (APCs) in the immune system and are central players of the adaptive immune response (Lee and Radford, 2019). DCs can be conventional (cDC) or plasmacytoid (pDC) based on their ontogeny (Wculek et al., 2020). According to DCs development, it might be immature or mature. Most immature DCs live on mucosal surfaces, while skin and solid organs operate as antigen sentinels. These DCs express fewer MHC I and II, T cell co-stimulating factors, and adhesion molecules (Wculek et al., 2020). Recent researches have illustrated that CAFs can influence the differentiation of monocytes into DCs as well as maturation, antigen presentation, and immune responses.

According to pertinent studies, CAF-derived TGF-β (Weber et al., 2005; Travis and Sheppard, 2014), VEGF (Gabrilovich et al., 1996; Huang et al., 2007) and inflammatory cytokine (Chomarat et al., 2000; Park et al., 2004) are reimplicated in restraining DCs function and maturation. TGF-β (Gabrilovich et al., 1996; Huang et al., 2007) mediates the downregulation of MHC class II molecules and costimulatory molecules in dendritic cells, inhibiting dendritic cell antigen presentation and activating the cytotoxic T-cell response. CAFs-produced IL-6 induces a tolerogenic phenotype in hepatocellular carcinoma DCs, increases tumor infiltration of immunosuppressive regulatory T cells (Tregs) (CD4CD25Foxp3), and decreases IFN-γ production by CD8 T cells (Cheng et al., 2016). Furthermore, tryptophan 2,3-dioxygenase (TDO2) secreted by CAFs inhibited the differentiation and function of DCs in a transplantable model of lung cancer, whereas TDO2 inhibition increased DC function and T cell responses (Silzle et al., 2004). In a recent study, WNT2 secreted by CAFs was found to inhibit the *in vitro* differentiation and immunostimulatory activity of DCs (Huang et al., 2022a). In primary OSCC tumors, WNT2 CAFs and CD8 T cells correlated negatively. Anti-WNT2 mAb restored anti-tumour T-cell responses and increased active DC in mouse OSCC and CRC syngeneic cancer models (Huang et al., 2022a). By inhibiting CAFs-derived WNT2, DC differentiation and antitumor T cell responses were restored. WNT2 produced by CAFs suppressed anti-tumor T cell responses mediated by DC *via* SOCS3/p-JAK2/p-STAT (110).

However, the detailed mechanisms by which CAF influences DCs development, maturation, and function are not yet understood, based on the existing evidence.

## 4 Interaction between CAFs and adaptive immune cells in the TME

### 4.1 Direct interaction between CAFs and T lymphocytes

When activated, naive CD4 T cells differentiate into various T helper (Th) subpopulations, the most common of which are Th1, Th2, and Th17 cells (Ruterbusch et al., 2020). CD4 Th1 cells mediate the immune response to viral infections and malignancies. In addition to antitumor CD8^+^ T cells, they are an important source of IFN-γ, which has direct antitumor effects (Zhu and Zhu, 2020). Th2 cells activate and maintain humoral or antibody-mediated immune responses against extracellular parasites, bacteria, allergens, and toxins (Zhu and Zhu, 2020). A predominantly Th1 response is associated with a better prognosis in human tumors such as colorectal, breast, brain cancers, whereas Th2 is linked to a worse prognosis for pancreatic and colorectal malignancies (Chraa et al., 2019).

Direct and indirect evidence suggests that CAFs recruit and balance CD4 effector T cell subsets to promote Th2 responses at the expense of Th1 responses. *In vivo* studies using the breast cancer 4T1 model depleted FAP CAFs by DNA vaccines led to increased expression of IL-2 and IL-7 (inducing Th1 cell differentiation) and decreased expression of IL-4 and IL-6 (inducing Th2 cell differentiation) in tumor homogenates, indicating that CAFs regulate the transition from Th1 to Th2 mediated immunity (Liao et al., 2009). TNF and IL-1 stimulate CAF in human pancreatic tumors to produce thymic stromal lymphopoietin (TSLP), which indirectly promotes Th2 cells by acting on dendritic cells. Specifically, TSLP increases Th2 recruitment through stimulated dendritic cell production of C-C motif chemokine ligand 17 (CCL17) and CCL2, naive CD4 T cells to polarize to the Th2 phenotype (measured by IL-13 production), thereby increasing Th2 numbers (De Monte et al., 2011). There is less evidence that CAF and Th17 interact, and it is less clear what role Th17 cells play in the immune system’s ability to fight cancer. In a recent study, CAFs in the colorectal area made RANTES and MCP-1 to attract Th17s instead of other T helper subpopulations (Su et al., 2010). This was made possible by TLR3 signaling to CAFs (Su et al., 2010). Also, CAFs helped naive CD4 T cells turn into Th17 cells by letting out IL-1, IL-6, IL-23, and TGFβ, which was partly caused by contact (Su et al., 2010). These studies demonstrate that CAFs may contribute to the establishment of a fraction of immunosuppressive helper T cells that is less cytotoxic.

Tregs serve a vital function in maintaining immune system homeostasis. Tregs interact positively with CAFs, cancer cells, and negatively with cytotoxic T lymphocytes (CTL) and natural killer cells (Ohue and Nishikawa, 2019). The possibility of interactions between CAFs and Treg cells has been demonstrated in numerous researches. In 2013, Kinoshita et al. (2013) revealed that the existence of Tregs coexisting with CAFs is correlated with poor lung adenocarcinoma patient outcomes. Sharma et al. (2005) reported a potential interaction between CAFs and Tregs *via* COX-2 production of CAFs in the lung or pancreatic cancer leading to their emission of PGE-2, which is known to stimulate Foxp3 expression. Liao et al. (2009) focused on FAP^+^ CAFs and showed that their depletion in breast tumors is connected with reduced Tregs Ozdemir et al. (2014) reduced *a*-SMA^+^ CAFs in pancreatic cancer animal models and detected an increase in CD4^+^FoxP3^+^ Tregs. In addition, CAFs excel in recruiting and inducing Tregs due to their strong secretory activity and capacity to release immunosuppressive cytokines. For instance, Chang et al. discovered that CCL5, a factor that CAFs can release, increases Tregs migration into tumors, and that blocking CCR5 signaling and knocking down CCL5 in tumor cells reduced Treg infiltration and slowed tumor progression in a mouse model of colorectal cancer (Chang et al., 2012). Other molecules such as Vascular endothelial growth factor A (VEGF-A) (Bourhis et al., 2021), C-C motif chemokine ligand 1 (CCL1) (Kuehnemuth et al., 2018), CCL2 (Kadomoto et al., 2021), CCL22 (Anz et al., 2015) and other factors have also been shown to be involved in promoting the recruitment and infiltration of Treg cells. CAFs not only increase Treg cells recruitment and infiltration, but they also promote their transformation and immunosuppression. For instance, in head and neck malignancies, TGFβ released by CAFs promotes T cells death and Tregs polarization, creating an immunosuppressive milieu (Takahashi et al., 2015). Julie Jacobs et al. (2018) examined the prognostic value of CD70 expression in CRC tissues using immunohistochemistry, as well as its interaction with fibroblast markers and Tregs. The involvement of CD70-positive CAF in migration and immune evasion was determined by *in vitro* experiments. Functionally, CD70-positive CAFs stimulated the frequency of naturally occurring Tregs and encouraged migration. Moreover, CAF altered the immunosuppressive TIL population of TME by secreting high levels of IL6, which decreased CD8 ^+^ TILs while increasing Foxp3^+^ TILs (Kato et al., 2018). Recently, using single-cell RNA sequencing, HuocongHuang et al. determined that antigen-presenting CAF (ap-CAF) originates from mesothelial cells and that ap-CAF induces naive CD4 T cells to form regulatory T cells in an antigen-specific transformation manner, induces Treg immunosuppressive function and suppresses CD8^+^ T cell function (Huang et al., 2022b).

All the above results indicate that CAFs may have an effect on Tregs in TME, leading to their recruitment and differentiation. Determining whether CAFs cause immunosuppression in the TME through interaction with Tregs and the precise mechanism of this process would undoubtedly contribute to a greater comprehension of the antitumor immune response.

CD8^+^ T cells, which are also known as CTLs, are responsible for mediating cytotoxic actions (St Paul and Ohashi, 2020). This is mostly done by making tumor cells go through a process called apoptosis, which is thought to be the most important part of antitumor immunity (St Paul and Ohashi, 2020).

Numerous studies indicate that CAF lowers the ability of CD8^+^ T lymphocytes to kill tumor cells by reducing T cell penetration into the tumor, preventing T cell trafficking in the microenvironment, and lowering cytotoxic activity (Freeman and Mielgo, 2020). When activated in the TME, CAF may block the recruitment of CD8^+^ T lymphocytes from the periphery to the tumor by secreting many cytokines and chemokines that result in immunosuppression. The CXCL12-CXCR4 chemokine axis is the most well-known of these mechanisms. T cells express the CXCL12 receptor, which FAPCAFs in the TME generate. This binding keeps TIL in the tumor stroma and stops them from getting to areas of the tumor with cancer cells (Wang et al., 2022). For instance, CXCL12 generation by activated CAF in PDAC increases migration of peripheral CD8^+^ T cells to active CAF in the peritumor stromal region, resulting in a concentration of CD8^+^ T cells in the pan-mesenchymal compartment and decreased recruitment to the tumor islets (Ene-Obong et al., 2013). In preclinical research on PDAC, inhibition of CXCR4 at both the pharmacological and genetic levels led to the rapid buildup of CD8^+^ T cells in tumors and a reduction in tumor growth (Feig et al., 2013). A new inflammatory CAF (iCAF) subpopulation was discovered in a recent study that used single cell RNA-sequencing to examine stromal cell populations in patients with TNBC(135). Genes in the CXCL12-CXCR4 chemoattractant pathway were turned up by iCAFs, and the presence of iCAFs was strongly linked to the failure and exclusion of CD8^+^ T cells (Kocher et al., 2020). Furthermore, CAF can reduce CD8^+^ T cell recruitment and inhibit their cytotoxic activity against tumor cells by releasing IL-6 and TGF-β. When co-inoculated with CAFs and tumor cells, Kato et al. demonstrated that tumor growth was more prominent in immunocompetent mice than immunodeficient nude mice, indicating that CAFs may aid tumor growth by modifying the immune response. Furthermore, the rejection of CD8^+^ T cells by CAFs is dependent on IL-6, and inhibiting IL-6 results in a substantial shift from FoxP3 to CD8^+^ T cells in the TIL population (Kato et al., 2018). It has been demonstrated that increased CAF-derived TGF-β secretion is associated with decreased CD8^+^ T cell accumulation in metastatic colorectal cancer and urothelial cancer as well as diminished sensitivity to immune checkpoint medications (Thomas and Massagué, 2005; Tauriello et al., 2018). Inhibition of CAF-derived TGF-β enhanced T cell density inside the tumor parenchyma in both of these malignancies, restored the effectiveness of checkpoint inhibition, and decreased metastatic burden.

In addition to CAF-secreted elements that directly influence CD8^+^ T cell control of migration and function in TME, it has been discovered that dense ECM structures can impede CD8^+^ T cell intratumoral migration (Vyas and Demehri, 2022).

### 4.2 CAFs inhibit T lymphocyte infiltration through ECM

The ability of activated CAFs to abnormally deposit ECM proteins such as fibronectin, collagen, and hyaluronan as well as matrix breakdown enzymes is a crucial characteristic of these cells (Tian et al., 2019). This prevents T-cell contact-dependent death of tumor cells in several solid cancers. CAFs were found to reduce the number of tumor-infiltrating CD8^+^ T lymphocytes by altering ECM composition. It was found that CD8^+^ T cells tend to gather in the stromal parts of these tumors, which have a much sparser network of fibrin and collagen fibrils than the islets of the tumor, which are surrounded by dense networks of collagen and fibrin fibers that run in parallel. The same result was found in ovarian cancer (Bougherara et al., 2015). CAFs made from activated tissue-resident stellate cells kept CD8^+^ T lymphocytes away from the tumor in stromal compartments in human tissue sections and a mouse model of pancreatic cancer, which was linked to a shorter survival time (Ene-Obong et al., 2013). In the same way, focal adhesion kinase, non-receptor tyrosine kinases, and enhanced stromal collagen 1 deposition were shown to be activated in pancreatic cancers, which led to inadequate CD8^+^ cytotoxic T cell infiltration. In mouse pancreatic cancer, blocking focal adhesion kinase 1 increased the number of CD8^+^ T cells and decreased the amount of collagen and CAFs (Jiang et al., 2016). NADPH oxidase 4 (NOX4), a TGFβ-1 downstream target that generates reactive ROS, can regulate the transition of fibroblasts to myofibroblasts. In a mouse model of CAF-rich tumors, silencing NOX4 or blocking it with drugs prevents TGFβ-driven differentiation of CAF into myofibroblasts and downregulates functional markers of fully differentiated CAF, such as αSMA and collagen 1 (141). Tumor hypoxia is one more element that affects how many solid tumors have less CD8^+^ T cell infiltration (Mortezaee and Majidpoor, 2021). In response to hypoxia, CAFs release a variety of angiogenic factors, including VEGF, which decreases the expression of cell adhesion molecules on endothelial cells, such as intercellular cell adhesion molecule (ICAM)-1/2 (Pietras and Ostman, 2010). Insufficient cell adhesion molecules, it is hard for peripheral CD8^+^ T-cells to move through the vascular system and reach the tumor site (Slaney et al., 2014).

### 4.3 CAFs inhibit T lymphocyte function by upregulating the expression of immune checkpoint molecules

Lower effector function and reduced proliferative capacity are two characteristics of antitumor T cell dysfunction, which is partly brought on by the overexpression of immunological checkpoint molecules (Lakins et al., 2018). CAFs have the ability to directly kill CD8^+^ T lymphocytes and express immunological checkpoint molecules such as PD-L1 and PD-L2 in their cells. (Lakins et al., 2018). Lakins et al. (2018) found that by increasing both PD-L2 and Fas ligands, CAFs from mouse lung adenocarcinoma and melanoma tumors can directly kill tumor-specific CD8^+^ T-cells, which helps the tumor stay alive. Fibroblasts from biopsies of melanoma patients show upregulation of both PD-L1 and PD-L2, which bind to the PD-1 receptor and directly counteract CD8^+^ T cell function (Khalili et al., 2012). In the same context, it was discovered that PD-L1 and PD-L2 were elevated in pancreatic cancer CAF expression and that CAF encourages the development of co-suppressive immune checkpoint receptors (such as PD-1) in proliferating T cells, which results in T cell malfunction (Gorchs et al., 2019). Melanoma-associated fibroblast-derived soluble factors significantly downregulated CD69 expression on the surface of activated CD8^+^ T cells and reduced granzyme B production and release. Melanoma-associated fibroblasts (MAFs) were also discovered to selectively dysregulate many immunological checkpoint regulators on CD8^+^ T cells. The MAF-derived soluble factors increase the number of BTLA-positive and TIGIT-positive CD8^+^ T cells by a large amount, interfere with intracellular CTL signaling, and make CTL less effective (Érsek et al., 2021). However, the underlying mechanism by which CAF promotes upregulation of suppressive immune checkpoints on CD8^+^ T cells is not fully understood.

### 4.4 CAFs inhibit T lymphocyte function by interfering with antigen presentation

It has also been demonstrated that CAFs suppress the T cell receptor (TCR) to decrease the proliferation and function of CD8 T cells, hence interfering with the antigen detection and activation process. For example, both *in vitro* and mouse models of pancreatic cancer showed that CAF-derived βig-h3 protein (also known as TGF-βi) had an inhibiting effect on tumor-specific CD8 T cells by acting directly on them (Goehrig et al., 2019). βIg-h3 interacts with CD61 on CD8^+^ T cells, causing Hic-5 protein to bind to Y505-phosphorylated Lck and inhibit TCR signaling, which inhibited tumor-specific CD8^+^ T-cell activation (Goehrig et al., 2019). Additionally, preventing professional APC and/or interfering with antigen expression can indirectly prevent CD8^+^ T cells from performing their cytotoxic activity. CAFs stop DCs or NK cells from presenting antigens by interfering with their normal development. They also cause immunosuppressive subpopulations and immune checkpoint expression, which makes it harder for effector T cells to fight tumors. For example, human hepatocellular carcinoma-derived CAFs can secrete IL-6, which upregulates the activation of the STAT3 signaling pathway in DCs and creates a regulatory DC (rDC) phenotype that can’t prime and activate T lymphocytes (Cheng et al., 2016).

### 4.5 CAFs inhibit T lymphocyte function through metabolic reprogramming

In recent years, there has been a growing body of data indicating CAF metabolic reprogramming provides an additional mechanism for inhibiting cytotoxic T cell function inside TME. For instance, it has been demonstrated that purinergic nucleosides produced by tumor-educated MSCs isolated from human cervical cancer (CeCa) patients inhibit the proliferation, activation, and effector activities of CD8^+^ T lymphocytes (de Lourdes Mora-García et al., 2016). Glycolytic CAFs release lactate to act on CD4^+^ T cells, decreasing Th1 and increasing Tregs (Comito et al., 2019). Because cancer cells are able to utilise lactate and pyruvate that is generated by CAFs, the activity of effector T cells is inhibited without an impact on the survival of cancer cells. This is because CAFs that glycolyze glucose diminish ambient glucose levels in the TME (Comito et al., 2019). The presence of CAFs inhibits the activity of CTL and reveals a vital role for arginase. In melanoma, CAF-derived soluble factors boost l-arginase activity and CXCL12 release, while also decreasing the amount of CD69 seen on activated CTL. The absence of CD8^+^ T lymphocytes in solid tumors may be due to the high levels of CXCL12 produced by CAFs, which might function as a chemoattractant and explain this phenomenon (Érsek et al., 2021).

In conclusion, CAFs promote immune suppression in the TME by promoting the cancer-promoting phenotype shift of naive T cells by boosting the function of immune inhibitory T lymphocytes, dampening the activity of effector T lymphocytes, interference with antigen presentation and metabolic reprogramming. There is a dearth of research into the impact of T lymphocytes on CAFs, which could be an exciting new area of study.

### 4.6 Interaction between CAFs and MDSCs

In addition of influencing immune responses to tolerate tumors, MDSCs support a number of neoplastic progression-related processes, including tumor angiogenesis, cancer stemness, and metastatic spread (Khaled et al., 2013).

Clinical data shows that CAFs play an immunosuppressive role associated with MDSCs. By releasing different cytokines and chemokines in the TME, CAFs can change how MDSCs are recruited and activated. Earlier studies have described the effect of CAF-secreted CXCL16 on monocyte aggregation in triple-negative (TN) breast cancer (Allaoui et al., 2016). Many studies have shown that CAFs can activate the signaling pathway molecule STAT3 by secreting IL6, leading to the conversion of monocyte precursors into MDSCs (Kim et al., 2012; Mace et al., 2013). Recent studies on esophageal squamous cell carcinoma, for instance, have underlined the significance of CAF-secreted IL-6 in the production of MDSCs and demonstrated that CAF-derived exosome-filled microRNA-21 (miR-21) also generates mononuclear MDSCs (M-MDSCs) by activating STAT3 signaling (Zhao et al., 2021). CXCL12 in the tumor microenvironment is mostly produced by CAF and plays a crucial function in attracting myeloid cells and supporting an immunosuppressive phenotype; inhibition of CXCL12 or its receptor CXCR4 lowers the amount of MDSC in the tumor (Obermajer et al., 2011; Feig et al., 2013). It was discovered that CAFs from hepatocellular carcinoma impact T cell development and function, as well as the patient’s overall longevity, by enticing monocytes to the TME by releasing CXCL12 and driving them to transform into MDSCs by activating STAT3 through IL-6 (Deng et al., 2017). Moreover, CAF, which is a significant source of CCL2, can also activate the STAT3 signaling pathway to increase MDSCs recruitment and promote tumor progression. Xuguang Yang et al. used a mouse liver tumor model to show that FAP CAFs are a major source of CCL2 and that CAF increases MDSCs recruitment *via* STAT3-CCL2 signaling to promote tumor growth (Yang et al., 2016b). A recent study found that LSCC-derived CAFs promote CCR2+ monocyte recruitment by secreting CCL2, which polarizes monocytes to the MDSCs phenotype, thereby inhibiting CD8^+^ T cell proliferation and IFN-γ production. The CAFs-MDSCs axis effect could be eliminated by inhibiting CCR2 and scavenging ROS, elucidating a potential therapeutic pathway to reverse CAF-mediated immunosuppression (Xiang et al., 2020). In addition to increasing MDSCS recruitment and activation to promote an immunosuppressive phenotype, a recent study showed that CAFs can regulate MDSCs function to enhance cancer stemness. The results of the study showed that CAF increased the expression and activity of 5-lipoxygenase (5-LO) in MDSCs by secreting IL-6 and IL-33, which greatly contributed to the efficiency of tumor sphere formation and stemness marker gene expression in intrahepatic cholangiocarcinoma (ICC) cells (Lin et al., 2022).

## 5 CAF targeting strategies

With further research and understanding of CAF-mediated suppression of the immune response, there has been a revival of interest in targeting CAFs for treatment in recent years. As shown previously, it is now well established that CAFs can play immunomodulatory roles: they can promote tumor progression directly or indirectly by increasing the content of suppressive immune cells and counteracting effector immune cell functions; and by participating in ECM remodeling and metabolic reprogramming to create an immune tolerance microenvironment. Because of their role in creating an immunosuppressive microenvironment during tumor growth, CAFs are now being considered potential therapeutic targets for the treatment of cancer and have also demonstrated the potential of CAF-targeting strategies in combination with immunotherapy. Table 1 briefly summarizes the CAF-targeted therapeutic strategies in clinical and preclinical studies. In clinical and preclinical studies, Table 1 gives a brief summary of the current ways that CAFs are treated. Different strategies are being investigated, including CAF activation and functional suppression, direct CAF depletion, combined with CAF-induced ECM remodeling limitation.

**TABLE 1:** CAF-related targeting modalities in preclinical or clinical studies.

Suppressing CAF activation and function by targeting associated effector molecules							
Target	Drugs name	Mechanisms	Combination therapy	Therapeutic effects	Cancer models	Status	Ref
Vitamin Ametabolism	All-trans retinoic acid (ATRA)	Retinol levels restoration, PSC de-activation	Gemcitabine	Increases T-cell infiltration	PDAC	Phase Ib	Kocher et al. (2020)
Vitamin D receptor	Calcipotriol	PSC de-activation	Gemcitabine	Revers chemoresistance	PDAC	Preclinical	Sherman et al. (2014)
Hedgehog	Sonidegib	Inhibits hedgehog signaling through SMO inhibition	Docetaxel	Inhibits tumor growth	TBNC	Phase I/II	Ruiz-Borrego et al. (2019)
NOX1/4	GKT137831 (setanaxib)	Inhibition and of CAFs formation	None	Increases T-cell infiltration	CRC	Preclinical	Ruiz-Borrego et al. (2019)
TGF-βR2/PD-L1	M7824	TGF-βR2 inhibition	None	Reduces tumor infiltration Tregs inhibits tumor development	PDAC	Preclinical	Ravi et al. (2018)
TGFBR1	Galunisertib	Prevents CAF activation and immunosuppression	Gemcitabine	Prolongs patients’ survival with minimal added toxicity	PDAC	Phase I/II	Herbertz et al. (2015)
TGF‐β	Fresolimumab	Neutralizing TGF‐β	None	Extend overall survivability	Breast cancer	Phase II	Formenti et al. (2018)
CXCR4	AMD3100 (plerixafor)	Inhibit CXCL12 production; prevents signaling from CAFs to immune cells	Anti-PD-L1 therapy	Promotes T-cell accumulation and eliminates cancer cells	CRC and PDAC	Preclinical	Biasci et al. (2020)
CCL2-CCR2	PF-04136309	CCL2-CCR2 signaling axis inhibition	FOLFIRINOX	Restricts immune suppression and improves clinical prognosis	PDAC	Phase Ib	Nywening et al. (2016)
IL-6	Tocilizumab	IL-6-JAK/STAT3 inhibition	None	Increases anticancer immunity	PDAC	Preclinical	Goumas et al. (2015)
JAK	Ruxolitinib	JAK-STA3 inhibition	Capecitabine	Inhibits tumor-promoting inflammation	Metastatic pancreatic cancer	Phase II	Hurwitz et al. (2015)
WNT2	WNT2 monoclonal antibody	WNT2 inhibition	PD-1 monoclonal antibody	Restore anti-tumor T cell reaction and increase the number of active DC	OSCC and CRC	Preclinical	Huang et al. (2022a)
Depleting CAFs directly by targeting surface markers							
FAP enzyme inhibition	DNA vaccine	Inhibits FAP enzymatic activity	Doxorubicin	Increase CD8T cell infiltration	CRC	Preclinical	Loeffler et al. (2006)
	OMTX705		Pembrolizumab	Increased infiltration of CD8^+^ cytotoxic T cells	PDAC	Preclinical	Fabre et al. (2020)
	^177^Lu-FAP-2286		None	Activation of immune cells	CRC	Phase I	Baum et al. (2022)
*a*-SMA-targeted nanoparticles	Cellax	Inhibits *a*-SMA enzymatic activity	None	Increases anti-stromal action and tumor inhibition	PDAC and breast cancer	Preclinical	Murakami et al. (2013)
Targeting CAF-derived ECM proteins							
;LOX	BAPN	LOX and LOXL inhibition	None	Anti-crosslinking and inhibites tumor growth and metastasis	Breast Cancer	Preclinical	Levental et al. (2009)
LOXL2	Simtuzumab	LOXL2 inhibition	None	Anti-crosslinking and inhibites tumor growth and metastasis	Breast Cancer	Preclinical	Grossman et al. (2016)
Hyaluronic acid	PEGPH20	Tumor stromal hyaluronan-targeted depletion	Nab-paclitaxel/Gemcitabine	Prolongs patients’ survival with less systematic side effect	PDAC	Phase II	Hingorani et al. (2018)
FAK	VS-4718	FAK inhibition	None	Decreases fibrosis and immunosuppressive cell populations	PDAC	Phase I	Jiang et al. (2016)

### 5.1 Suppressing CAF activation and function by targeting associated effector molecules

CAF exhibits phenotypic flexibility, and that certain of its phenotypes may be tumor suppressive, according to recent research. As a result, it is possible to reprogram CAF to a dormant or even immunologically licensed phenotype.

Ene-Obong et al. (2013) discovered that in PDAC, quiescent PSC suppressed malignant progression of PDAC cells by upregulating apoptosis and suppressing Wnt-linked protein signaling through the expression of secreted frizzled-related protein 4 (SFRP4). This finding raises the possibility of indirectly targeting malignant cells by controlling tumor-tumor crosstalk. Notably, co-engagement of all-trans retinoic acid (ATRA) with PSC quiescence has been demonstrated to boost intratumoral CD8^+^ T cells and improve survival. ATRA and gemcitabine-nab-paclitaxel combination therapy has been tried in phase I clinical studies and is currently being investigated in phase Ib trials (Kocher et al., 2020). The vitamin D receptor (VDR), which regulates PSC transcriptional activity and can convert PSC into a dormant state, is likewise a viable therapeutic approach for targeting CAFs (Sherman et al., 2014). In mice with spontaneous PDAC, calcipotriol, a ligand of the VDR family, enhanced chemotherapy response and decreased tumor development. It also decreased inflammation and fibrosis in mouse pancreatitis and the interstitium of human tumors (Sherman et al., 2014). Cazet et al. (2018) investigated the TNBC model and discovered that Hedgehog-dependent CAF activation and ECM remodeling supported the establishment of CSC ecotopes, hence resulting in docetaxel resistance. This work suggests that treatment strategies targeting the Hedgehog pathway are feasible. In phase I and phase II clinical trials with an inhibitor of the hedgehog signaling system (sonidegib) in conjunction with docetaxel, three out of twelve TNBC patients with distant metastases benefited from the combination therapy (Ruiz-Borrego et al., 2019). The NOX1/4 inhibitor GKT137831 (setanaxib) stops or turns around CAF differentiation in tumors. It also helps CD8^+^ T-cells move into tumors and overcomes anti-PD-1 therapies (Ford et al., 2020).

As mentioned above, CAFs promote immune escape by secreting relevant effectors, so targeting important CAF-related effector molecules as well as signaling pathways seems to be a more practical strategy to slow down the development of CAF and immune cell contacts during immunosuppression-induced TME. Several preclinical and clinical investigations have demonstrated that targeting the immune functions of CAF might boost immunotherapeutic efficacy.

The activation of CAF and the interaction of CAF with immune cells are both significantly influenced by TGF-β (Angioni et al., 2021). It makes sense to target this pathway by inhibiting TGF-β, which has been done using a number of methods, such as TGF-β mRNA-directed agents, antibodies, fusion proteins, and small molecule kinase inhibitors against TGFBRs (Ciardiello et al., 2020). For example, poor anti-PD-L1 efficacy in advanced uroepithelial carcinoma has been associated with a facilitative effect of TGF-β1 signaling on CD8^+^ T-cell tumor exclusion. Combined administration of TGF-β blocking antibodies and anti-PD-L1 antibodies on mouse tumor models improved treatment responsiveness and improved T cell infiltration in tumors (Mariathasan et al., 2018). In preclinical investigations, bifunctional fusion proteins that target PD-L1 and TGF-β have demonstrated anticancer efficacy. The anti-CTLA4-TGFβ-R2 molecule is more effective than ipilimumab (an anti-CTLA-4 antibody), at inhibiting tumor progression and decreasing tumor-infiltrating Treg cells (Ravi et al., 2018). Galunisertib, a TGFBR1 kinase inhibitor chosen for its reasonably safe toxicological profile, started clinical trials more than a decade ago (Herbertz et al., 2015). In phase I studies, intermittent dosing was demonstrated to be well-tolerated, suggesting a therapeutic window. A number of clinical trials have since tested the safety of galunisertib alone or in combination with other chemotherapeutic drugs, with acceptable findings (Ciardiello et al., 2020; Kim et al., 2021). A monoclonal antibody called fresolimumab is capable of neutralizing all three TGFβ isoforms. High-dose fresolimumab led to a longer OS than low-dose fresolimumab when used in conjunction with radiation in patients with breast cancer who had distant metastases (Formenti et al., 2018). It is clear from the above that FAP-derived CXCL12 inhibits the accumulation of CD8T cells in tumors in the tumor microenvironment (Feig et al., 2013). By inhibiting the CXCL12-CXCR4 signaling pathway, AMD3100 (plerixafor), a CXCR4 inhibitor, has been shown to diminish CAF-mediated immunosuppression and boost the response to anti-PD-1 immunotherapy (Biasci et al., 2020). In the mouse breast cancer models, genetic deletion of CXCR4 in stromal cells expressing the αSMA marker decreased fibrosis, increased T-cell infiltration, and enhanced sensitivity to checkpoint inhibition (Chen et al., 2019). The CXCL-CXCR2 axis was inhibited, which inhibited tumor angiogenesis and prolonged survival. The infiltration of MDSCs, neutrophils, and ARG-1^+^ TAMs was significantly reduced, while apoptotic tumor cells and iNOS^+^ M1-like TAMs increased significantly (Sano et al., 2019). CCR2 inhibitor (PF-04136309) dramatically decreased the number of tumor-infiltrating macrophages and Treg cells while boosting the number of effector T lymphocytes in the TME, consequently enhancing pancreatic cancer antitumor immunity (Nywening et al., 2016). Additionally, there are treatment approaches that target the key pathways triggered by CAF, such as the IL6-JAK1/2-STAT pathway (Mace et al., 2013). Tocilizumab, a humanized anti-IL-6R monoclonal antibody, shown substantial anticancer effects in numerous forms of cancer in preclinical tests (Goumas et al., 2015). In a double-blind phase II study of the JAK1/JAK2 inhibitor ruxolitinib in conjunction with capecitabine, patients with MPC (multiple primary malignancies) exhibited improved tolerability and a longer life expectancy (Hurwitz et al., 2015). Recently, it was found that using WNT2 monoclonal antibody with PD-1 monoclonal antibody significantly restored intra-tumor anti-tumor T cell responses and improved anti-PD-1 efficacy in mouse OSCC and CRC homologous tumor models by increasing the number of active DCs (Huang et al., 2022a).

### 5.2 Depleting CAFs directly by targeting surface markers

Currently, CAF is eliminated primarily by targeting CAF surface markers such as FAP, *a*-SMA, and PDFR. Although CAF marker inhibitors are the primary kind of CAF depletion therapy, the absence of CAF-specific markers and the adverse effects of this method have hampered their practical application.

In mouse models of colon and breast cancer, it has been shown that killing FAP^+^ CAFs by CD8^+^ T lymphocytes prevents primary tumor development and metastasis (Lo et al., 2015). In a pioneering investigation, an oral DNA vaccine targeting FAP targets was shown to drastically decrease primary tumor cell proliferation and neovascularization, considerably boost intra-tumor medication absorption, and minimize metastasis in colon and breast cancers by killing tumor-associated fibroblasts (Loeffler et al., 2006). Although FAP-specific CAR-T cells can all produce limited anti-tumor effects, they also produce serious side effects, such as bone marrow toxicity (Lee et al., 2022). OMTX705 is a first-in-class antibody–drug conjugate (ADC) that targets the tumor microenvironment with FAP-dependent cytotoxic activity. It has showed effectiveness in mouse models and a spectrum of solid malignancies, with the potential to increase CD8^+^T cell infiltration and activity when paired with therapeutically relevant medications (Fabre et al., 2020). FAP-2286 is a therapeutic and diagnostic agent comprised of a FAP-binding peptide with high affinity for human FAP protein and radionuclide labeling. In initial clinical studies, it showed promise for ameliorating the unpleasant symptoms of invasive adenocarcinoma and was well tolerated (Baum et al., 2022). Docetaxel conjugate nanoparticules that target αSMA stromal cells in a mouse model of breast cancer suppress the growth of metastases (Murakami et al., 2013). In contrast, in a pancreatic cancer model using mice transgenic for αSMA thymidine kinase, elimination of αSMA myofibroblasts resulted in more aggressive tumors and decreased animal survival (Özdemir et al., 2014). In addition, myofibroblast-depleted mice showed suppressed immune surveillance with elevated CD4 Foxp3 Tregs along with resistance to anti-CTLA-4 immunotherapy (Rhim et al., 2014). It is evident from the above that CAF does not fully express either *a*-SMA or FAP, which poses a significant obstacle to the precise strategy of CAF-based therapy.

### 5.3 Targeting CAF-derived ECM proteins

CAF-rich tumor Stroma is abundant in collagen, fibronectin, and proteoglycans, which encase and restrict the mobility of T cells (Evanko et al., 2012). Consequently, several CAF-targeted treatments reduce the associated fibrotic products and restrict ECM remodeling by inhibiting the fibrotic signal pathway. The modified ECM mitigates the inhibitory influence of the TME on tumor recruitment of immune effector cells and increases anti-tumor immunity (McCarthy et al., 2018). The LOX and LOXL enzymes were the target of preclinical investigations involving *ß*-aminopropionitrile (BAPN), which greatly decreased tumor fibrosis and restored cellular function, but their toxicity precluded their use in clinical trials (Belhabib et al., 2021). In preclinical trials, simtuzumab suppressed tumor cell proliferation and metastasis by targeting LOXL2, which regulated the structure of collagen; nevertheless, clinical trials were unsuccessful (Grossman et al., 2016). Enzymatic hydrolysis of HA is an additional method for decreasing tumor interstitial pressure (TIFP). To improve intratumoral medication delivery and synergistic tumor therapy. Based on preclinical research, anti-HA therapy using the vascular enzyme hyaluronidase was employed to destroy tumor cells by lowering TIFP, boosting vascular permeability, and promoting gemcitabine transport efficiency (Maloney et al., 2019). In a similar way, combining PEGPH20 with nab-paclitaxel/gemcitabine increased PFS in untreated patients with metastatic PDAC, especially those with high levels of HA (189). FAK inhibitors target integrin signal transduction. By reducing FAK signaling pathway activation, VS-4718 lowers fibrosis and immunosuppressive cells and increases pancreatic tumor sensitivity to chemotherapy and immunotherapy (Jiang et al., 2016). Although certain medicines targeting fibrosis-activated signaling pathways and fibrosis-related products had promise in preclinical models, they failed to enhance survival in patients with multiple advanced malignancies in clinical trials.

### 5.4 Discussion on CAF targeting strategy

However, many of the current strategies for targeting CAFs as immunotherapy have been clinically ineffective. First, as previously mentioned, the lack of specific markers for CAF means that current CAF-targeted therapies must address the insurmountable challenge of simultaneously improving antitumor efficacy and reducing systemic side effects. Second, most of the current preclinical studies have been conducted with overall CAFs, with insufficient understanding of the functions of CAFs in tumors, ignoring their high heterogeneity in tumor development. It has been demonstrated that different subtypes of CAFs have different functions in tumor immune regulation because of the high heterogeneity of CAFs. For example, pCAFs (Cancer-promoting CAFs) inhibit antitumor immunity mainly by expressing FAP-α or *a*-SMA multiplex, while rCAFs (Cancer-restraining CAFs) inhibit pancreatic cancer growth with Meflin (glycosylphosphatidylinositol-anchored protein) as a biomarker (Takahashi et al., 2021). The discovery of the tumor suppressive function of specific subgroups of CAFs provides a potential explanation for the failure of clinical trials targeting immunotherapy with CAFs. Therefore, preclinical studies are needed to determine whether the targeted subpopulations of CAFs function as fully immunosuppressed before applying immunotherapeutic strategies for CAFs. For example, the transcriptome and proteome of relevant CAF subgroups were studied in depth using technologies such as single-cell sequencing to analyze the immunosuppressive functions of specific CAFs to identify the exact targeting mediators. In addition, CAFs cells are more susceptible to phenotypic transformation than tumor cells and exist in a complex tumor microenvironment, so standard methods for cancer drug screening may be less applicable to CAFs. Therefore, suitable *in vivo* and *in vitro* models are needed to screen for effective targets of CAFs. These challenges may be the reason why CAF-targeted therapies are rarely put into clinical use. Future research on these issues still needs to be intensively studied to find more precise and effective molecular targets for CAFs.

## 6 Conclusion and outlook

In recent years, evidence showing the role and significance of CAFs in tumorigenesis, progression, immunosuppression, and drug resistance in a variety of malignancies has increased. As key components of the TME, CAFs are closely associated with the TME as well as the whole host in a context-dependent manner with phenotypic and functional heterogeneity. This paper illustrates the relationship between CAFs and immune cells, where CAFs not only directly influence the activity of immune cells, but also alter the ECM within TME and induce metabolic reprogramming. The interaction between cancer-associated fibroblasts and immune cells has a significant impact on the regulation of tumor growth.

**FIGURE 2:**
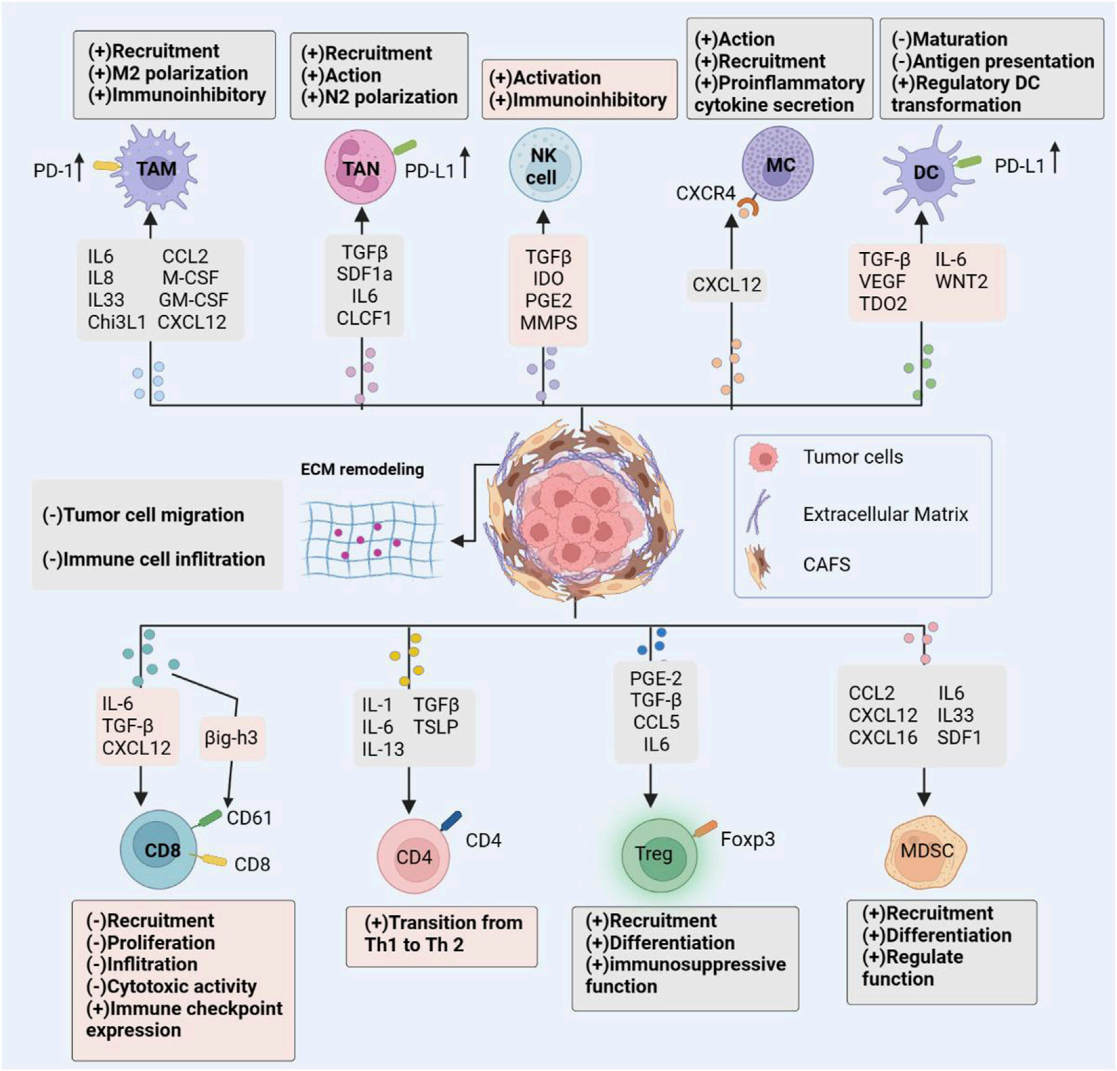
CAF immune Effects. Cancer-associated fibroblasts interact with the immune microenvironment in tumors, influencing the majority of immune cell populations and resulting to an immunosuppressive tumor microenvironment. By secreting an array of chemokines, cytokines, and other effector molecules, CAFs modulate immune cell-mediated antitumor immunity. Promoting the recruitment, activation, and immunosuppressive effects of immunosuppressive cells; limiting the cytotoxic activity and cytokine production of effector immune cells such as natural killer (NK) cells and cytotoxic T lymphocytes (CTL).

As previously described, this review summarizes the mechanism of interaction between CAFs and immune cells in TME. CAFs induce the formation of immunosuppressive microenvironment by secreting a series of cytokines that have direct effects on immune cells, specifically by inducing the recruitment and activation of TAMs, TANs, CD4T cells, tregs, MCs, MDSCs cells; inducing TAMs, TANs cells polarization to an immunosuppressive phenotype; and inhibition of CD8T cell and NK cell activity and function. In addition, CAFs can also secrete ECM proteins to induce ECM remodeling to inhibit CD8T cell infiltration; upregulate the expression of immune checkpoint molecules to directly kill CD8 T cells; inhibit TCR to hinder CD8 T cell proliferation and activation; and metabolic reprogramming to inhibit CD8 T cell proliferation and activity, thus indirectly leading to an immunosuppressive microenvironment. Although research on CAFs is expanding, there are still significant gaps in the therapeutic efficacy of targeted CAFs in clinical trials. The main CAF targeting strategies currently available are through the following pathways: 1) inhibition of CAF activation and function by targeting relevant effector molecules; 2) direct depletion of CAF by targeting surface markers; and 3) targeting CAF-derived ECM proteins. The mechanism of interaction between CAFs and immune cells is summarized in Figure 2. Numerous pre-clinical studies failed to detect any appreciable anti-tumor effects or significantly increase patient survival. The clinical failure of CAF-targeted therapy may be due to the following reasons; lack of specific markers and systemic side effects of targeted drugs; ignoring the highly heterogeneous nature of CAFs, with some subpopulations belonging to tumor suppressor phenotypes, targeting overall CAFs cannot achieve good clinical results; inapplicable drug screening methods; difficulty of *in vitro* models to restore the complex tumor microenvironment conditions.

Future research focusing on the heterogeneity of CAFs is needed to identify the functions and specific mechanisms of specific subpopulations of CAFs through single cell sequencing to elucidate their clinical importance and impact on cancer development in order to find effective targeted immunotherapies and mediators. Second, the complicated tumor microenvironment (hypoxia, acidic microenvironment, anomalies in tumor vascularity, etc.,) necessitates the employment of appropriate *in vivo* and *in vitro* study models that are more useful for research. Current research foci on CAFs in carcinogenesis and treatment resistance are primarily focused on subgroup analyses and functional studies because the majority of solid tumors have many CAFs subgroups.
